# The TOPAZ study: a home-based trial of zoledronic acid to prevent fractures in neurodegenerative parkinsonism

**DOI:** 10.1038/s41531-021-00162-1

**Published:** 2021-03-01

**Authors:** Caroline M. Tanner, Steven R. Cummings, Michael A. Schwarzschild, Ethan G. Brown, E. Ray Dorsey, Alberto J. Espay, Nicholas B. Galifianakis, Samuel M. Goldman, Irene Litvan, Nijee Luthra, Nikolaus R. McFarland, Kyle T. Mitchell, David G. Standaert, Douglas C. Bauer, Susan L. Greenspan, James C. Beck, Kenneth W. Lyles

**Affiliations:** 1 Department of Neurology, Weill Institute for Neurosciences, University of California San Francisco, San Francisco, CA USA; 2 Parkinson’s Disease Research Education and Clinical Center, San Francisco Veteran’s Affairs Health Care System, San Francisco, CA USA; 3 San Francisco Coordinating Center,California Pacific Medical Center Research Institute, Departments of Medicine and Epidemiology & Biostatistics, University of California San Francisco, San Francisco, CA USA; 4 MassGeneral Institute for Neurodegenerative Disease, Department of Neurology, Massachusetts General Hospital, Boston, MA USA; 5 Center for Health + Technology and Department of Neurology, University of Rochester Medical Center, Rochester, NY USA; 6 James J. and Joan A. Gardner Family Center for Parkinson’s Disease and Movement Disorders, Department of Neurology, University of Cincinnati, Cincinnati, OH USA; 7 Division of Occupational and Environmental Medicine, University of California San Francisco, San Francisco Veterans Affairs Health Care System, San Francisco, CA USA; 8 Parkinson and Other Movement Disorders Center, Department of Neuroscience, University of California San Diego, La Jolla, CA USA; 9 Norman Fixel Institute for Neurological Diseases, Department of Neurology, University of Florida College of Medicine, Gainesville, FL USA; 10 Department of Neurology, Duke University School of Medicine, Durham, NC USA; 11 Department of Neurology, University of Alabama at Birmingham, Birmingham, AL USA; 12 Departments of Medicine and Epidemiology & Biostatistics, University of California San Francisco, San Francisco, CA USA; 13 Osteoporosis Prevention and Treatment Center, Department of Medicine, University of Pittsburgh, Pittsburgh, PA USA; 14 Parkinson’s Foundation, New York, NY USA; 15 Department of Medicine, Duke University School of Medicine, VA Medical Center, Durham, NC USA

**Keywords:** Phase IV trials, Parkinson's disease

## Abstract

The Trial of Parkinson’s And Zoledronic acid (TOPAZ, https://clinicaltrials.gov/ct2/show/NCT03924414) is a unique collaboration between experts in movement disorders and osteoporosis to test the efficacy of zoledronic acid, an FDA-approved parenteral treatment for osteoporosis, for fracture prevention in people with neurodegenerative parkinsonism. Aiming to enroll 3,500 participants age 65 years or older, TOPAZ is one of the largest randomized, placebo-controlled clinical trials ever attempted in parkinsonism. The feasibility of TOPAZ is enhanced by its design as a U.S.- wide home-based trial without geographical limits. Participants receive information from multiple sources, including specialty practices, support groups and websites. Conducting TOPAZ in participants’ homes takes advantage of online consent technology, the capacity to confirm diagnosis using telemedicine and the availability of research nursing to provide screening and parenteral therapy in homes. Home-based clinical research may provide an efficient, convenient, less expensive method that opens participation in clinical trials to almost anyone with parkinsonism.

## Introduction

**Fig. 1:**
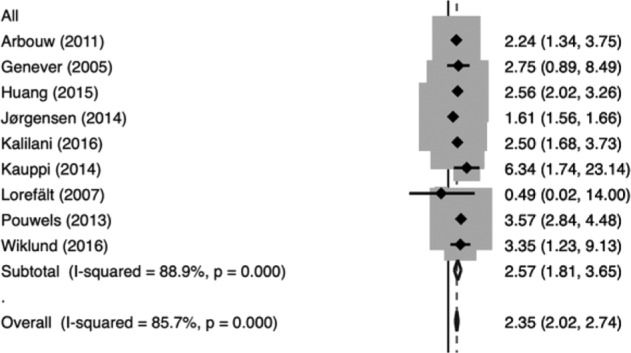
Meta-analysis of the Association of Hip Fracture in People with Parkinson’s Disease. The forest plot shows effect sizes and 95% confidence intervals for individual studies, showing an overall estimated increased risk of hip fracture, determined from health records or by self report, in people with Parkinson’s disease. Adapted from Schini et al 2020^1^ with permission.

People with Parkinson’s disease (PD) have a high risk of fracture that may be due, in large part, to their high risk of multiple falls and impaired reflexes to protect against injury during a fall. A recent systematic review and meta-analysis found that people with PD, based on data from health systems and self-report, have a 2.35-fold increased risk of hip and 1.8-fold increased risk of all fractures besides vertebral fractures^1^ (Fig. 1). The relative risk was somewhat greater for men than women and was not confined to those with advanced disease. The consequences of fractures – sustained disability and death – are also likely to be much greater for people with PD^2^. Patients with other forms of neurodegenerative parkinsonism, such as progressive supranuclear palsy and multiple system atrophy, have similar risk of falls and fractures, and similar barriers to treatment^3,4^. For the purpose of this manuscript, we will refer to the group of disorders including PD and other neurodegenerative parkinsonisms as “parkinsonism”. For the vast majority of people with parkinsonism who are age 65 or older, the risk of fracture may be sufficient to warrant treatment with medications. Bisphosphonate therapy has been shown to reduce fracture risk in older women who have osteoporosis defined as a low bone mineral density (BMD), with less conclusive but suggestive evidence in men, and might be useful for fracture prevention in parkinsonism^5,6^.

Anecdotally, only a very small proportion of people with parkinsonism receive treatment to reduce fracture risk. There are several barriers to treatment of people with parkinsonism^7^. First, there is no evidence that treatments reduce the fracture risk of people with parkinsonism whose increased risk may be due to multiple falls. Second, the conventional approach to assessment with BMD testing and medical visits for interpretation, prescription of osteoporosis treatment, and follow-up to assess response to treatment is not familiar to neurologists and can be burdensome for people with parkinsonism. Additionally, compliance with oral treatments for osteoporosis is notably poor. It may be worse for older people with parkinsonism who are taking treatments for parkinsonism and other conditions^8^.

The Trial Of Parkinson’s And Zoledronic acid (TOPAZ) was designed to overcome these barriers. Using a randomized, placebo-controlled, double blind design, TOPAZ aims to determine whether treatment that improves BMD and reduces fractures in people with osteoporosis or hip fracture also reduces fracture risk in people with parkinsonism. It also tests a novel approach to treatment: that any person with parkinsonism age 65 or older without contraindications to treatment would receive treatment without referral for evaluations or BMD testing. To overcome limited adherence with treatments, participants are treated with one intravenous infusion of zoledronic acid, a treatment that improved BMD and reduced fractures in postmenopausal women with osteoporosis^9^ and in men and women after a hip fracture^10^. Its effects on BMD and fracture risk persist for at least two years^11–13^. For the convenience of people with parkinsonism, and to reduce barriers to treatment, zoledronic acid can be administered by a nurse in the patient’s home^13^.

## Results and discussion

TOPAZ is a unique collaboration between neurologists expert in movement disorders and internists expert in osteoporosis to examine the efficacy of a treatment to prevent one of the most common causes of disability in parkinsonism, fractures. TOPAZ is the largest double blind randomized controlled clinical trial ever attempted in people with parkinsonism. Importantly, TOPAZ is a direct to consumer trial – participants need not be receiving care in a specialty clinic and may enroll, be assessed and treated completely from home. Participation within the US is not restricted by geography and recruitment is done from many sources. Conducting TOPAZ in participants’ homes takes advantage of the technologies to consent participants online, the capacity to perform telemedicine visits to confirm the presence of parkinsonism, and the availability of research nursing to conduct medical screening and give parenteral therapy in homes.

The need for bone mineral density testing provides an additional barrier to treatment in the parkinsonism population. We extensively considered whether to require BMD testing before enrollment in TOPAZ, but our analyses of the HORIZON trial data found that efficacy of zoledronic acid for fracture risk reduction was the same regardless of a person’s BMD. This has been confirmed by the randomized trial of zoledronic acid in older women who had a *T*-score above −2.5^14^. Additionally, the consent process emphasizes that potential participants may opt to have BMD testing and seek evaluation and treatment instead of participating in the trial. If we demonstrate a reduction in risk of fractures, then the implication for clinical practice would be that zoledronic acid would be recommended to all patients with Parkinson’s disease or parkinsonism age 65 or older, women or men, who have no contraindication to the treatment, without need for BMD testing.

Remote neurologic assessments of people with PD using video software have been shown to be valid and acceptable supplements to in-patient assessments for decades^15,16^, but widespread use has been limited by poor reimbursement and unfamiliarity of patients and doctors. Although the COVID-19 pandemic temporarily stopped almost all in-person visits to neurology practices, patient ‘visits’ are continuing using telemedicine video software, such as Zoom or Skype. Traditional outpatient practices have rapidly converted to remote assessments and legal and reimbursement barriers have been reduced. The IPMDS has identified the importance of widespread implementation of telemedicine to reduce the risk of COVID-19 exposure for people with parkinsonism and their Telemedicine Study Group has developed resources to facilitate widespread use of this technology by patients and doctors^17^. Remote educational activities such as webinars and podcasts can highlight TOPAZ as a completely home-based research opportunity at a time when most clinical trials participation is severely limited. Increased familiarity of patients with telemedicine as a result of these pandemic-associated changes, combined with steadily growing internet access among older U.S. residents (https://www.pewresearch.org/internet/fact-sheet/internet-broadband/), might facilitate the online enrollment and telemedicine examinations for TOPAZ.

As familiarity with clinical care for parkinsonism using telemedicine grows, this approach to conducting clinical research and, specifically, clinical drug trials, may provide an efficient, convenient, and less expensive method that opens participation in clinical trials to almost any person with neurodegenerative parkinsonism, unlimited by their location. The successful implementation of an at home-based treatment protocol, without a requirement for BMD testing, as utilized in TOPAZ, will reduce barriers to the clinical implementation of study results. Further, the TOPAZ trial will fill important knowledge gaps about the utility of an established fracture-preventative therapy in treating an at-risk population, potentially identifying an intervention that could greatly reduce morbidity and mortality in parkinsonism.

## Methods

TOPAZ is a home-based trial. Also called ‘virtual’, or ‘direct-to-participant’ trials, this type of trial is conducted over internet and in participants’ homes^18–20^. Participants provide electronic informed consent and enroll via a study website. Clinical evaluations can be performed by telemedicine and by research nurses visiting the participant’s home. Oral vitamin D can be sent by express mail and parenteral study drug can be administered at home by a research nurse. Study outcomes can be assessed by electronic medical record (EMR), telemedicine, or patient questionnaire or interview. The Parkinson’s Foundation hosts the TOPAZ website that describes the trial and allows potential participants to begin screening, consent, and enrollment (https://www.parkinson.org/research/TOPAZ-Trial).

The TOPAZ study has been approved by the Western Institutional Review Board (WIRB).

The primary aim of TOPAZ is to test the efficacy of an infusion of zoledronic acid, 5 mg, compared with placebo, to reduce the risk of clinical fractures by conducting a randomized, double-blind, placebo controlled clinical trial. A secondary aim is to evaluate reduction in the risk of hip fracture. Zoledronic acid reduced total mortality, not attributable to prevention of fractures, in two, but not all trials^21^. Consequently, a secondary aim is to test whether treatment with zoledronic reduces total mortality.

### Eligibility criteria

People with parkinsonism who are age 65 or older are eligible for TOPAZ. The enrollment criteria for TOPAZ are intended to be broad to include almost everyone with parkinsonism. TOPAZ only excludes people with parkinsonism who are bed and wheelchair bound (equivalent to Hoehn and Yahr Stage V^22^), those who have had a hip fracture (because zoledronic acid has been proven to reduce fracture in such patients), are currently receiving bisphosphonate therapy or have contraindications to zoledronic acid including renal insufficiency (based on FDA guidelines) or lesions of the oral mucosa that may predispose to osteonecrosis of the jaw, a rare side effect of the drug. At the home visit the nurse assesses renal function using a finger-stick test for estimated glomerular filtration rate and examines the participant’s mouth for oral lesions. TOPAZ does not exclude people with parkinsonism with dementia or cognitive impairment because they have an increased risk of fracture and might benefit from zoledronic acid. Potential participants who have dementia and are unable to complete enrollment and consent may participate by having a court-approved power-of-attorney (POA) or legally authorized representative (LAR) complete the enrollment questions and informed consent on the participants’ behalf.

### Sample size

TOPAZ will be the largest double-blind placebo-controlled randomized clinical trial ever conducted in people with parkinsonism. TOPAZ plans to enroll 3500 participants who are age 65 years or older. Each of the endpoints will be analyzed using Cox proportional hazards regression. This number of participants will provide the trial 90% power to detect a 25% reduction in the risk of any fracture, a clinically important reduction that is similar to the approximately 30% reductions seen in osteoporosis trials^9,23^. Power will be 80% to detect the secondary end point of a 40% reduction in hip fracture and the exploratory end point of a 28% reduction in mortality.

### Recruitment

TOPAZ is recruiting participants using multiple methods. Movement disorder specialists affiliated with the Parkinson’s Study Group (PSG, http://www.parkinson-study-group.org/) or Parkinson Foundation Centers of Excellence and non-specialist neurology groups that care for people with parkinsonism in large public hospital settings, such as Cook County Hospital in Chicago, are informing potentially eligible people with parkinsonism about TOPAZ. Each of the practice groups provide information during clinic visits, including the URL for the study website. The study website provides information about TOPAZ. Potential participants have the opportunity to answer screening questions to determine eligibility and provide informed consent for the next steps in the trial. In addition, practice groups may contact patients prior to their visit, by mail, email or telephone, to briefly inform them about the study and set aside time at the visit to learn more. Participants are offered the opportunity to access the study website by computer or tablet in the clinic, and if requested, clinic staff can provide assistance. Many practices have lists of their age-eligible patients and can provide a link to the TOPAZ website by email or post. Some groups participate in community fairs to provide information and promote research where information about TOPAZ or a table with computer tablets open to the TOPAZ website may reach people with parkinsonism who do not have a visit to the practice scheduled during the recruitment period.

Recruitment for TOPAZ is not limited to neurology practices. The Parkinson’s Foundation is contacting potential participants who have interacted with their HelpLine and given permission to contact them about research opportunities. They are sending emails that include a link to the TOPAZ website (https://www.parkinson.org/research/TOPAZ-Trial) and a YouTube video that describes the TOPAZ trial (https://tinyurl.com/td4ujr4). In addition, neurology practices are providing information to patient support groups, including newsletter articles and speakers at meetings. Other online resources such as listings of clinical trials, Fox Trial Finder, podcasts, and social media will also be utilized.

### Electronic interactive informed consent

**Fig. 2:**
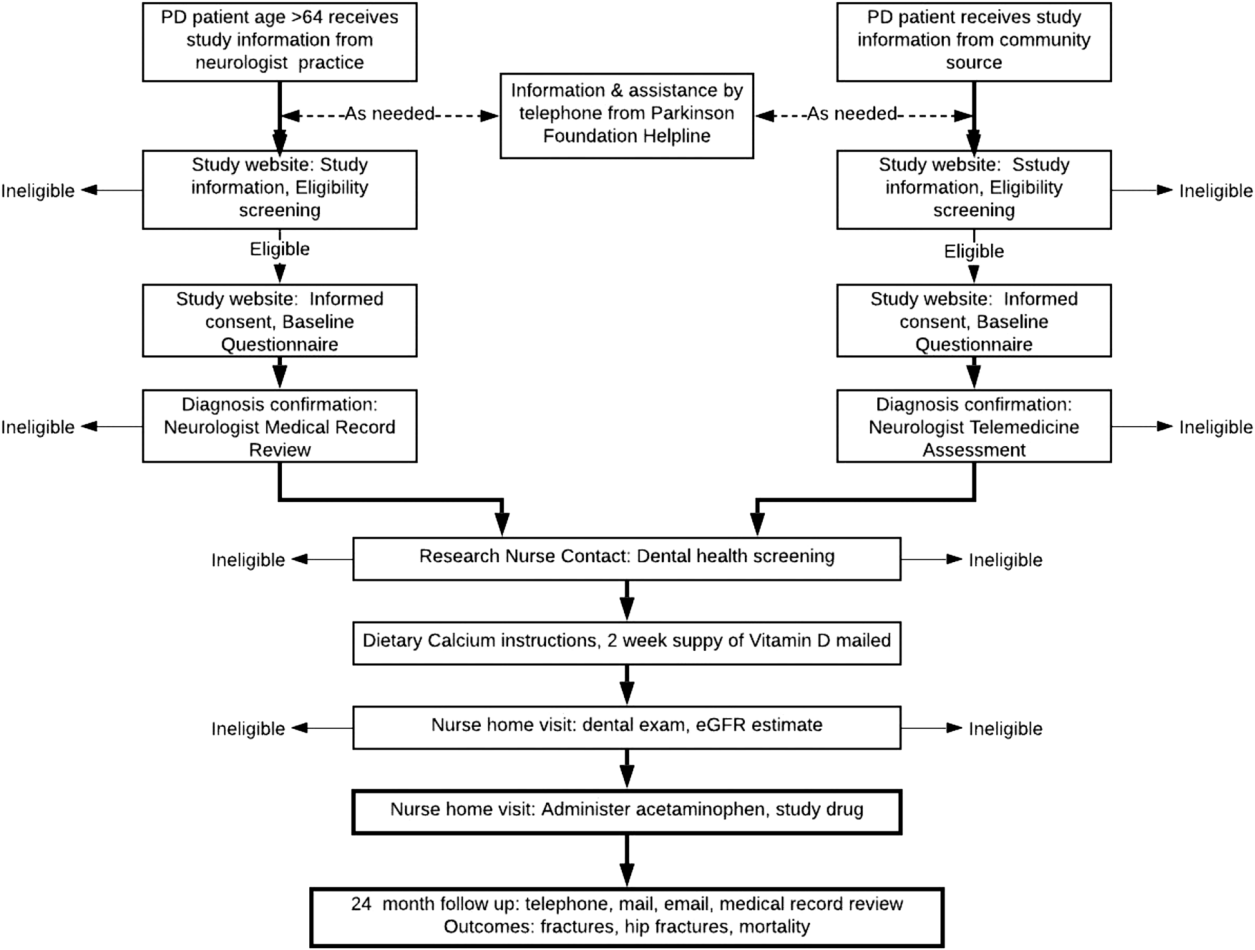
Flow of Participation in TOPAZ Study. Participants may self-refer or be referred by neurologists. Informed consent is provided online and treatment is provided in the home. Followup isconducted remotely.

Potential participants provide informed consent online, via the TOPAZ website. Known as electronic consent, or eConsent^13^, the process begins with a description of the TOPAZ study, followed by reading the consent form, and electronically ‘signing’ the form using the Docusign system (Fig. 2). To enroll, the participant must answer a few simple multiple choice questions to demonstrate their understanding of the trial. For example, one question reinforces that the participant may seek clinical assessment and treatment for potential osteoporosis instead of enrolling in the trial. As the goal is to improve the participant’s understanding of the trial, a wrong answer triggers a pop-up of the section of the consent that describes the corresponding part of the consent and the participant has another chance to answer the question. Participants also have the opportunity to speak with nurses familiar with the study through the toll-free Parkinson’s Foundation Help line (https://www.parkinson.org/Living-with-Parkinsons/Resources-and-Support/Helpline). A description of the consent process is available online (https://youtu.be/F9exePu9Chk). This approach to obtaining consent has been shown to improve participants’ understanding of trials compared with a paper consent process^19,24,25^. For participants who have a POA or LAR, the representative may provide and sign the consent on behalf of the participant. In addition to consent, the participant provides approval to obtain medical records to confirm parkinsonism diagnosis and disease features, the occurrence of fracture and death.

### Confirmation of the diagnosis of parkinsonism

After participants provide online consent, the TOPAZ Neurology Core confirms diagnosis in two separate ways, depending on how the participant was recruited into the study. For participants who are patients of physicians in a participating PSG or Parkinson Foundation Center of Excellence neurology practice, the diagnosis of parkinsonism is confirmed by medical record review. Following informed consent, the participant’s neurologist determines whether PD or another form of neurodegenerative parkinsonism is present^26^ and provides additional information, including age at parkinsonism onset. For participants who are not patients at participating sites, the Neurology Core conducts an online diagnostic assessment using telemedicine.

The telemedicine diagnostic assessment is performed by a PSG neurologist using a standardized evaluation, conducted by computer or mobile phone based video assessment, as recommended by the International Parkinson Disease and Movement Disorder Society Telemedicine Study Group (https://www.movementdisorders.org/MDS/About/Committees-Other-Groups/Telemedicine-in-Your-Movement-Disorders-Practice-A-Step-by-Step-Guide.htm). Assessments include versions of the Unified Parkinson’s Disease Rating Scale and the Montreal Cognitive Assessment validated for remote use^27,28^. Participants with a confirmed diagnosis of PD or another form of neurodegenerative parkinsonism^26^ are eligible to continue.

### In home study drug delivery

Eligible participants are contacted by a research nursing service to provide pre-medication and schedule a home visit by a research nurse. All participants receive two weeks of vitamin D supplements, 800 IU per day, to take just prior to the nurse home visit to prevent the very low risk of hypocalcemia after the infusion. Participants also receive recommendations for adequate calcium in their diet that are consistent with recommendations of the National Osteoporosis Foundation.

A particularly unique feature of TOPAZ is the medical screening and delivery of the intravenous study drug in the participant’s home. Research nurse services are certified to conduct clinical trial procedures in homes including the parenteral administration of study drugs. TOPAZ has partnered with PCM trials (https://pcmtrials.com/), a national organization of nurses that provides research services for over 90% of the population of the United States. PCM Trials manages the study drug, including storage, blinding, randomization, and delivery to the research nurse to take the intravenous setup to the participant’s home. To confirm eligibility to receive the study drug, the nurse calls the participant to schedule a home visit for study drug infusion and asks a series of questions about dental health. If there are no problems, the visit is scheduled. At the home visit, the nurse collects information on falls, and conducts an oral examination to assure that there are no lesions that would predispose to the development of osteonecrosis of the jaw, a rare occurrence after administration of zoledronic acid. If the dental exam is questionable, the nurse uses a secure smart phone protocol to send a picture of the mouth to the Medical Safety Officer at Duke University where is it immediately reviewed for eligibility. To prevent the rare occurrence of worsening renal function that might happen in participants with existing renal insufficiency, the nurse tests the participant’s creatinine with a validated “Stat-sensor” device that automatically calculates the participant’s estimated glomerular filtration rate (eGFR). A participant is excluded for an eGFR level of 35 ml/min or lower. If the participant has neither exclusion, the nurse administers the study drug – zoledronic acid or placebo – over 45 min. This rate is slower than the standard 15 min infusion time in order to reduce the chance that the participant will have an “acute phase reaction” that occasionally follows the administration. The reaction, characterized by low grade fever, generally mild arthralgia, myalgia, and/or headache, starts within 24 h and generally resolves by 72 h. To further minimize the chance and severity of the symptoms, the nurse administers 1 gram of acetaminophen by mouth prior to the infusion. The participant is instructed also to take acetaminophen every 6 h for 24 h, and if symptoms occur, to continue until symptoms resolve. The combination of very slow infusion and prophylactic acetaminophen is estimated to reduce the chance of an acute phase reaction to about 10% following zoledronic acid^13^.

Although there are no data suggesting that zoledronic acid or the transient acute phase reaction would influence symptoms or progression of parkinsonism, the first 175 participants who complete the telemedicine assessment and are randomized will be assessed again four months after blinded treatment to test for potential effects on parkinsonism features. To reduce variability, the same neurologist will perform the initial and follow-up assessments.

A Medical Safety Officer and a Neurology Safety Officer will be available through a 24-hour Study Helpline. Research nurses and participants will be instructed to call the Study Helpline with unexpected symptoms, or other concerns or study questions. Because of the rare risks of atypical femur fractures or osteonecrosis of the jaw after treatment with ZA, we will also advise participants to contact their physician or the Helpline if they experience new and persistent thigh pain or a sore in their mouth lasting ≥2 weeks. Health issues reported to the Helpline will be recorded as adverse events.

### Endpoint assessment

TOPAZ uses two approaches to achieve complete follow-up for the occurrence of fractures and deaths. In the first approach, conducted successfully by the San Francisco Coordinating Center in previous fracture endpoint studies, trial participants or their proxies are contacted by the Coordinating Center every four months by postcard, telephone, or email, to ask about the occurrence of a fracture or one or more falls during that interval. A report of a fracture prompts collection of data about date and location of medical care to obtain objective documentation of the fracture in a radiology report or discharge summary. In the second method, for participants who originated from health systems, the system performs an annual search of the EHRs for diagnoses of fracture or occurrence of death, along with collection of X-ray reports or discharge summaries confirming the event.

At the completion of the study, results will be shared with participants and, if requested, treatment assignment will be shared.

### Reporting summary

Further information on research design is available in the Nature Research Reporting Summary linked to this article.

## Supplementary information

Reporting summary

## Data Availability

No datasets were generated or analyzed during the current study.
